# Adaptive Vectorial Restoration from Dynamic Speckle Patterns Through Biological Scattering Media Based on Deep Learning

**DOI:** 10.3390/s25061803

**Published:** 2025-03-14

**Authors:** Yu-Chen Chen, Shi-Xuan Mi, Ya-Ping Tian, Xiao-Bo Hu, Qi-Yao Yuan, Khian-Hooi Chew, Rui-Pin Chen

**Affiliations:** Key Laboratory of Optical Field Manipulation of Zhejiang Province, Department of Physics, Zhejiang Sci-Tech University, Hangzhou 310018, China

**Keywords:** biological scattering media, convolutional neural network, vectorial reconstructions, Transformer

## Abstract

Imaging technologies based on vector optical fields hold significant potential in the biomedical field, particularly for non-invasive scattering imaging of anisotropic biological tissues. However, the dynamic and anisotropic nature of biological tissues poses severe challenges to the propagation and reconstruction of vector optical fields due to light scattering. To address this, we propose a deep learning-based polarization-resolved restoration method aimed at achieving the efficient and accurate imaging reconstruction from speckle patterns generated after passing through anisotropic and dynamic time-varying biological scattering media. By innovatively leveraging the two orthogonal polarization components of vector optical fields, our approach significantly enhances the robustness of imaging reconstruction in dynamic and anisotropic biological scattering media, benefiting from the additional information dimension of vectorial optical fields and the powerful learning capacity of a deep neural network. For the first time, a hybrid network model is designed that integrates convolutional neural networks (CNN) with a Transformer architecture for capturing local and global features of a speckle image, enabling adaptive vectorial restoration of dynamically time-varying speckle patterns. The experimental results demonstrate that the model exhibits excellent robustness and generalization capabilities in reconstructing the two orthogonal polarization components from dynamic speckle patterns behind anisotropic biological media. This study not only provides an efficient solution for scattering imaging of dynamic anisotropic biological tissues but also advances the application of vector optical fields in dynamic scattering environments through the integration of deep learning and optical technologies.

## 1. Introduction

The interaction of biological scattering media with incident optical fields has long posed a significant challenge in optical imaging, remaining an interesting research topic. Dynamic particles and structural inhomogeneity within biological tissues induce multiple complex scattering events, which severely degrade image quality [1]. This scattering causes phase distortion and uneven light intensity distribution, complicating image reconstruction. In particular, owing to the dynamic scattering nature of biological tissues, conventional optical imaging techniques face limitations in recovery imaging from dynamic speckle patterns generated after passing through biological scattering media. While various optical imaging techniques, including optical coherence tomography (OCT) [2,3,4], photoacoustic imaging (PAI) [5,6,7], and fluorescence microscopy [8,9], have been employed to address this, inherent limitations remain [10], especially for the restoration of dynamic speckle patterns. Chicken breast tissue slices can serve as an effective model for simulating the behavior of biological tissues in optical imaging and measurement due to their similarities in optical properties to human soft tissues [11,12,13]. The diverse components in chicken breast exhibit different absorption and scattering coefficients, contributing to both absorption and scattering during light propagation. Additionally, chicken breast tissue demonstrates dynamic characteristics, such as minor movements or deformations due to the evaporation of water during experiments, which can affect light propagation paths and scattering patterns, thereby exhibiting dynamic scattering properties. These complexities present significant challenges for traditional optical scattering models and imaging algorithms, requiring the robustness and generalization of an imaging model [14,15,16].

In recent years, deep learning-based imaging techniques have provided novel approaches for tackling the challenges associated with de-scattering [17,18]. By leveraging extensive training datasets, deep learning models can overcome certain limitations of traditional physical imaging techniques, especially in image reconstruction, noise reduction, and de-scattering [19,20]. Convolutional neural networks (CNN) have proven particularly effective in reducing scattering noise, enhancing contrast, and improving the quality of biological tissue imaging [21,22]. However, a conventional CNN, which depends on local receptive fields for extracting local features, struggles to capture global structural information. Moreover, as the depth of the network increases, the vanishing gradient problem hinders the model’s ability to learn high-quality global and detailed features, thereby constraining imaging performance [23].

In this work, we propose a deep learning-based polarization-resolved restoration to facilitate an accurate and efficient imaging recovery from dynamic speckle patterns that pass through biological scattering media. The utilization of two orthogonal polarization components in vector optical fields provides an additional dimension to significantly enhance the robustness and generalization for vectorial reconstructions of two orthogonal polarization components from a dynamic speckle pattern. Compared to our previous work for the reconstruction of speckle images in isotropic static scattering media (frosted glass) [24], this proposed network model is developed by combining a convolutional neural network (CNN) as the primary framework and the integration of Transformer architecture for global feature extraction to the biological tissues (chicken breast tissue slices), and the self-attention mechanism of the Transformer is utilized to capture global details within images [25,26]. This advanced learning-based polarization-resolved restoration technique can achieve the mapping relation between the dynamic speckle patterns and the input optical fields, providing a more accurate and efficient solution for biological tissue scattering imaging.

## 2. Deep Learning-Based Polarization-Resolved Restoration Model

### 2.1. Scattering Transmission Matrix for Vector Optical Fields

The propagation of optical fields within complex scattering environments is a highly intricate phenomenon. Upon traversing a scattering medium, the information conveyed by the outgoing light waves becomes disordered, and the scattering medium performs multiple encodings of both the incident optical field and its associated information. This enables the description of the process by which the incident field traverses the scattering medium through the establishment of a transmission matrix [27,28]. To characterize the transmission properties of vector light in scattering media, Tripathi et al. introduced the vector transmission matrix (VTM) in 2012 [29]. Generally, the relationship between the input vector optical field and the output vector optical field is expressed as follows:

(1) $$E_{out}=\left[\begin{array}{c} E_{out,x}\left(u,v\right)\\ E_{out,y}\left(u,v\right) \end{array}\right]=\sum_{m,n,u,v}\left[\begin{array}{cc} T_{11}\left(m,n,u,v\right) & T_{12}\left(m,n,u,v\right)\\ T_{21}\left(m,n,u,v\right) & T_{22}\left(m,n,u,v\right) \end{array}\right]\left[\begin{array}{c} E_{in,x}\left(m,n\right)\\ E_{in,y}\left(m,n\right) \end{array}\right],$$

where *T*[…] denotes the vector transmission matrix. Here, (m,n) and (u,v) represent the spatial coordinates of the input and output light fields, respectively, while $E_{in,x}\left(m,n\right)$, $E_{in,y}\left(m,n\right)$ and $E_{out,x}\left(u,v\right)$, $E_{out,y}\left(u,v\right)$ represent the complex amplitudes of the two orthogonal polarization components of the incident and outgoing vectors, respectively. Unlike scalar transmission matrices, vector transmission matrices not only describe the transmission characteristics of light intensity but also incorporate the polarization states of the light waves. This makes VTM crucial for studying the polarization behavior of light in inhomogeneous media. Different from a conventional isotropic scattering medium (e.g., ground glass) in which the two orthogonal polarization components propagate independently, and $T_{12}\left(m,n,u,v\right)=T_{21}\left(m,n,u,v\right)=0$ [24,29], the two orthogonal polarization components propagating in anisotropic biological tissues are reciprocally converted, i.e., $T_{12}\left(m,n,u,v\right)\neq 0$, and $T_{21}\left(m,n,u,v\right)\neq 0$. The anisotropic feature of a biological tissue complicates the evolution of the polarization state. On the other hand, it provides an extra dimension to effectively restore the speckle patterns especially for a scalar input light field (see the experimental reconstruction results below).

On the other hand, the biological tissues dynamically vary with minor movements or deformations due to the evaporation of water or gravity. Therefore, it is difficult to restore the polarization information from a speckle behind a biological tissue only by using a VTM. A deep learning network model with generalization and robustness is required for a more accurate and efficient solution for biological tissue scattering imaging [30]. Here, a network model is developed by combining CNN as the primary framework and the Transformer architecture for global feature extraction, to optimize the polarization information capture in scattering imaging.

### 2.2. Trans-CNN Network Architecture

**Figure 1:**
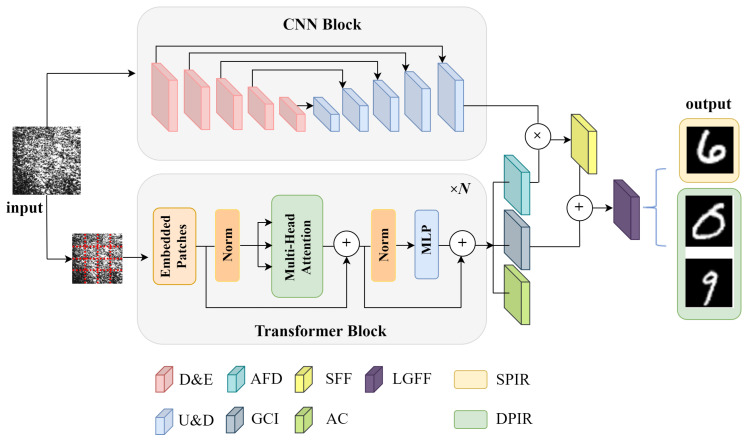
Diagram of the Trans-CNN network architecture. D&E: downsampling and encoder, U&D: upsampling and decoder, AFD: attention-based feature distributions, GCI: global contextual information, SFF: spatial feature fusion, AC: attention coefficients, LGFF: local–global feature fusion, SPIR: single-phase image reconstruction, DPIR: dual-phase image reconstruction.

Vector optical fields convey multidimensional information, including intensity, phase, and polarization states, offering enhanced spatial features compared to traditional scalar optical fields. To effectively capture the complex spatial characteristics and fully leverage this high-dimensional information for a more accurate image reconstruction, this study introduces a novel network architecture, termed the Trans-CNN network. This architecture combines the strengths of U-Net [30,31] and the Transformer model, enabling precise and robust imaging reconstruction of vector optical fields. The design of the Trans-CNN network is illustrated in Figure 1. This proposed network comprises two parallel encoding paths that independently process the input images, with each path extracting features from distinct perspectives. The U-Net path employs convolutional operations to focus on capturing detailed localized features in the speckle images, effectively extracting textural information from the local spatial domain. On the other hand, the Transformer path utilizes a self-attention mechanism to model long-range dependencies between pixels on a global scale, effectively addressing the complex polarization and phase information inherent in vector optical fields. This dual-path structure allows the network to effectively combine local and global feature extraction, enhancing its ability to reconstruct the high-dimensional spatial characteristics of vector optical fields for accurate imaging.

The U-Net architecture in the proposed network is designed with four submodules, each consisting of two convolutional layers followed by a pooling layer, which performs iterative downsampling to capture increasingly abstract features. In the encoder of the Transformer, the input image $I\in R^{H*W*C}$ is first divided into patches of size P×P. Each patch is then projected into a *K*-dimensional space, expressed mathematically as follows:

(2) $$X_{embed}=X*W_{proj}+pos_{emb},$$

where $X_{embed}$ represents the input image in the high-dimensional feature space, *X* denotes the pixel matrix of the input image, $W_{proj}$ denotes the projection matrix, and $pos_{emb}$ represents the positional embedding, which preserves the spatial location information of each patch. The patch embedding vectors, after integrating the positional embeddings, are input into the Transformer encoder. The Transformer encoder comprises multiple self-attention layers and feed-forward neural network layers, which are designed to capture the global information of the input image.

The images fed into the network are speckle images of size 256×256 pixels, accompanied by corresponding label images derived from processed MNIST handwritten digits. During the feature extraction phase, the features obtained from the CNN and Transformer paths are combined using multiplication and concatenation, resulting in a richer high-dimensional feature representation. When the network is employed for the reconstruction of a single image, i.e., the phase information carried by the two orthogonal polarization components are similar, the final output image retains the same dimensions as the input image. If the network aims to simultaneously reconstruct the distinct phase information associated with the two orthogonal polarization components in the vector optical field, the multi-channel features must be split into two independent channels in the final layer of the decoding path. Each channel corresponds to the phase information of one of the orthogonal polarization components, ensuring that the network effectively outputs and reconstructs the two-phase images.

## 3. Experimental Results

### 3.1. Generation and Data Acquisition of Vector Optical Fields

**Figure 2:**
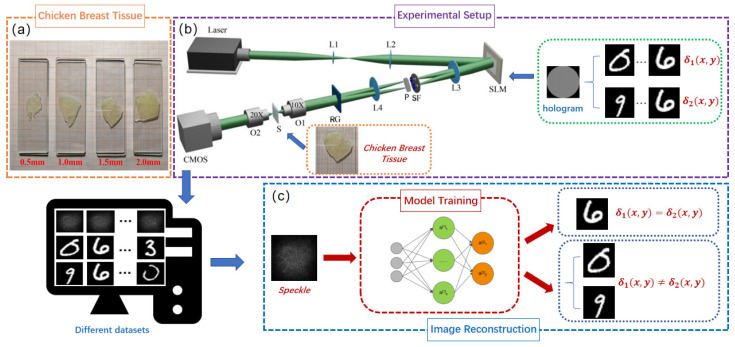
(**a**) Chicken breast tissue slices of varying thicknesses. (**b**) Schematic of the 4f optical system for generating vector optical fields and speckle patterns behind a chicken breast tissue. Key experiment components include a 532 nm laser source, a spatial light modulator (SLM), lenses L1–L4, microscope objectives O1 and O2, SF: a spatial filter, P: λ/4 (or λ/2) plate, RG: a Ronchi grating, S: biological tissue samples, and a CMOS camera. (**c**) The speckle patterns serve as inputs to the trained Trans-CNN for vectorially reconstructing one or two distinct phase images.

The vector optical fields are generated by using a 4f system, employing the coherent superposition of orthogonal left-handed and right-handed circularly polarized basis vectors, as well as orthogonal horizontally and vertically polarized basis vectors. Different thickness samples of chicken breast tissues (0.5 mm, 1.0 mm, 1.5 mm, 2.0 mm) are shown in Figure 2a. The experimental setup is illustrated in Figure 2b.

A solid-state laser with a wavelength of 532 nm illuminates a spatial light modulator (SLM, HoloEye-Pluto2) with a resolution of 1920×1080 pixels, using a beam-expanding collimating system composed of lenses L1 (focal length f=30 mm) and L2 (focal length f=250 mm). A two-dimensional computer-generated hologram (2D CGH) is loaded onto the SLM. The beam emerging from the SLM generates multiple diffraction orders in the *x* and *y* directions. The optical waves traverse lens L3 (focal length f=150 mm) and a spatial filter (SF) with dual apertures to select the +1 diffraction order in both *x*- and *y*-directions. Following spatial filtering, the optical waves are converted into orthogonal left-handed and right-handed circularly (or orthogonal horizontally and vertically) polarized light using a λ/4 (or λ/2) plate. Subsequently, the two beams are combined by a Ronchi grating (RG) to generate a vector beam. The resulting vector beam is focused onto a scattering medium *S* (fresh chicken breast tissue slices). For chicken breast tissues of varying thicknesses (0.5 mm, 1.0 mm, 1.5 mm, and 2.0 mm), the estimated values of absorption and scattering coefficients under room temperature conditions are as follows [32]:

- 0.5 mm thickness: absorption coefficient: 0.1∼0.3 ${\mathrm{cm}}^{-1}$, scattering coefficient: 50∼100 ${\mathrm{cm}}^{-1}$;
- 1.0 mm thickness: absorption coefficient: 0.2∼0.5 ${\mathrm{cm}}^{-1}$, scattering coefficient: 80∼150 ${\mathrm{cm}}^{-1}$;
- 1.5 mm thickness: absorption coefficient: 0.3∼0.7 ${\mathrm{cm}}^{-1}$, scattering coefficient: 100∼180 ${\mathrm{cm}}^{-1}$;
- 2.0 mm thickness: absorption coefficient: 0.4∼1.0 ${\mathrm{cm}}^{-1}$, scattering coefficient: 120∼200 ${\mathrm{cm}}^{-1}$.

The scattered light is subsequently collected through the objective lens and recorded as speckle patterns using a CMOS camera (DaHeng, Beijing Daheng Photoelectric Technology Co., Ltd., Beijing, China, MER2-302-56U3M). The two-dimensional hologram loaded onto the SLM is expressed by the transmittance function as follows:

(3) $$\begin{array}{c} t\left(x,y\right)=1+\frac{\gamma }{2}\cos\left(2\pi f_{0}x+\delta_{1}\left(x,y\right)\right)+\frac{\gamma }{2}\cos\left(2\pi f_{0}y+\delta_{2}\left(x,y\right)\right), \end{array}$$

where $f_{0}$ denotes the spatial frequency, γ represents the modulation depth, and $\delta_{1}\left(x,y\right)$ and $\delta_{2}\left(x,y\right)$ correspond to the additional phase distributions in the *x* and *y* directions, respectively.

In this experiment, the open-source dataset MNIST [33] of handwritten digit images was selected as the additional phase distributions $\delta_{1}\left(x,y\right)$ and $\delta_{2}\left(x,y\right)$, corresponding to the two orthogonal polarization components of the vector light field. These phase distributions were encoded into a two-dimensional hologram, which was then loaded into the SLM. Substantial speckle images were collected with a resolution of 2048×1536 pixels for image reconstruction. These images were resized to 256×256 pixels for input into the network model. A total of 10,000 speckle images and their corresponding label images were collected, and the dataset was appropriately divided into training and testing subsets at 8:2. During training, the speckle patterns served as network inputs, while the original handwritten digits acted as labels. Once the network training was completed, the actual experimentally acquired speckle patterns were directly input into the network to reconstruct the desired encoded images.

### 3.2. Adaptive Restoration of Dynamic Varying Speckle Patterns Through a Scattering Medium of Chicken Breast Tissue

**Figure 3:**
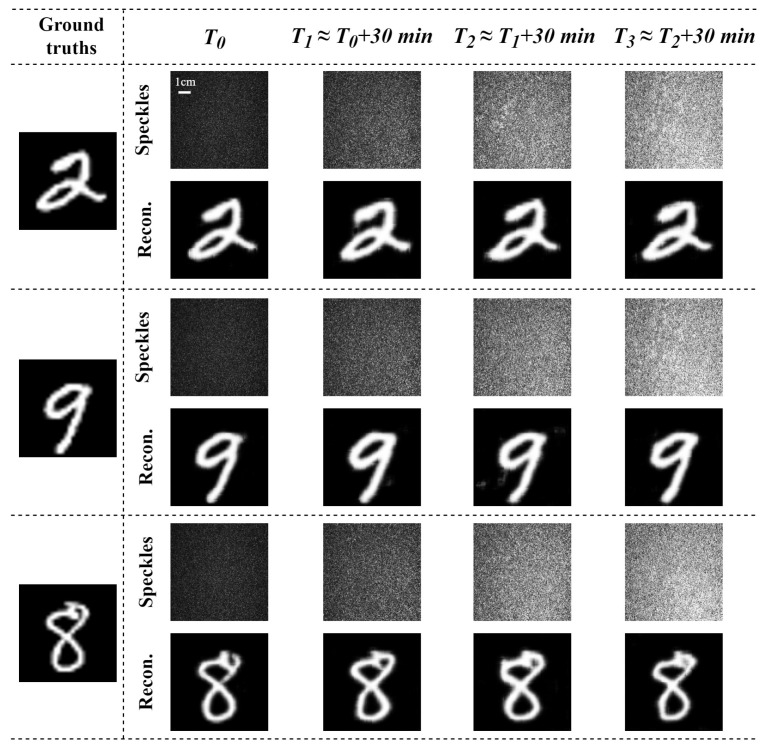
Temporal evolution of speckle image variations and the corresponding image reconstruction results.

The vectorial restoration of dynamic varying speckle patterns through a scattering medium of chicken breast tissue (1.0 mm thick) during different time periods was investigated. The two orthogonal polarization components of a vector optical field carry the same image information $\delta_{1}\left(x,y\right)=\delta_{2}\left(x,y\right)$, and the recovery results are shown in Figure 3. Based on our experimental observations of the water evaporation rate in chicken breast tissue slices, we found that the physical properties of the tissue undergo significant changes within a time frame ranging from several minutes to tens of minutes. To effectively capture these dynamic changes, we selected a 30 min interval for our experiments, which allowed us to observe and analyze the tissue’s behavior during the water evaporation process. The speckle patterns behind a chicken breast tissue across four different time periods corresponding to the same MNIST image varied significantly, due to the dynamic scattering properties of a chicken breast tissue slices over time. The experiment reconstruction results indicate that the proposed method can restore the corresponding ground truths even when the speckle patterns are dynamically varying, although the restored results slightly differ from each other, Here, we quantitatively evaluated the reconstruction results using the Pearson correlation coefficient (PCC), structural similarity index (SSIM), and peak signal-to-noise ratio (PSNR) [34,35,36] as evaluation metrics for the reconstructed images. The results are summarized in Table 1. The results demonstrate that the robustness and generalization of the Trans-CNN network significantly enhances the model’s adaptability to achieve the mapping relations for dynamically varying datasets, effectively improving the reconstruction performance.

### 3.3. Image Reconstruction Using Scalar and Vector Optical Fields Through Scattering Media of Chicken Breast Tissues

**Figure 4:**
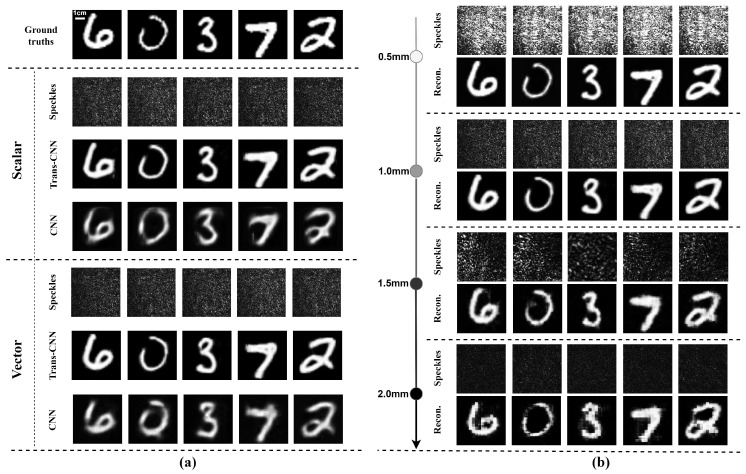
Reconstruction of single-phase images with Trans-CNN and CNN networks through chicken breast tissue slices using a scalar or vector optical field. (**a**) Feconstructed results with Trans-CNN and CNN networks through 1.0 mm thick chicken breast tissue slices using a scalar or vector optical field; (**b**) reconstruction results with Trans-CNN network through varying thicknesses of chicken breast tissue slices using a vector optical field.

The image reconstruction results with the proposed Trans-CNN network and conventional convolutional neural network (CNN) are shown in Figure 4. The image reconstruction results obtained from these two different networks under identical conditions indicate that the network incorporating the Transformer architecture is more effective in recovering the global structural information of images. In complex scattering environments, the self-attention mechanism of the Transformer captures long-range pixel dependencies, thereby preserving additional image details. In contrast, the conventional CNN primarily relies on local feature extraction, resulting in an inferior reconstruction of global structures and producing blurred edges and structural noise in the reconstructed images.

In addition, the reconstruction of a single image by a vector optical field with two orthogonal polarization components, i.e., the two orthogonal polarization components of a vector optical field carrying the same image information $\delta_{1}\left(x,y\right)$ and $\delta_{2}\left(x,y\right)$, was examined compared to the reconstruction of a single image by a scalar optical field, with the same network architecture, data processing techniques, and training methods.

The restored images clearly demonstrate that the reconstructed images derived from vector optical fields are sharper, with greater detail, as shown in Figure 4a. In contrast, the reconstructed images derived from scalar optical fields exhibit inferior edge detail and texture, whereas vector optical fields are more effective in preserving these features. The comprehensive utilization of two orthogonal polarizations within vector optical fields significantly enhances the robustness against noise in complex scattering environments. As shown in Table 2a, the vector optical field outperforms the scalar optical field in terms of the PCC, SSIM, and PSNR, indicating superior reconstruction image consistency, structural information, and lower noise levels. These varying thicknesses of chicken breast tissue slices were employed to further evaluate the image recovery performance of vector optical fields with the Trans-CNN model. The four datasets with thicknesses of 0.5 mm, 1.0 mm, 1.5 mm, and 2.0 mm of chicken breast tissue were constructed, and each dataset consisted of 10,000 pairs of speckle images and their corresponding label images. The comparison of the reconstructed images demonstrates the impact of varying scattering medium thicknesses on the image quality, as illustrated in Figure 4b. The image reconstructed using a 0.5 mm thick medium was the clearest, exhibiting the most detail. As the thickness increased, the image details gradually became more blurred, with the 2.0 mm thick medium yielding the poorest reconstruction and showing a significant loss of detail information.

### 3.4. Reconstruction of Dual-Phase Images of Orthogonal Polarization Components Passing Through Chicken Breast Tissues with Various Thicknesses

Different from the recovery of single-phase image information $\delta_{1}\left(x,y\right)$ = $\delta_{2}\left(x,y\right)$, the reconstruction of dual-phase images $\delta_{1}\left(x,y\right)\neq \delta_{2}\left(x,y\right)$ carried by the two orthogonally polarized components of a vector optical field passing through chicken breast tissues was investigated. The two phase images carried by the two orthogonal polarization components from a single speckle pattern behind the chicken breast tissue were simultaneously reconstructed with various thickness of samples.

**Figure 5:**
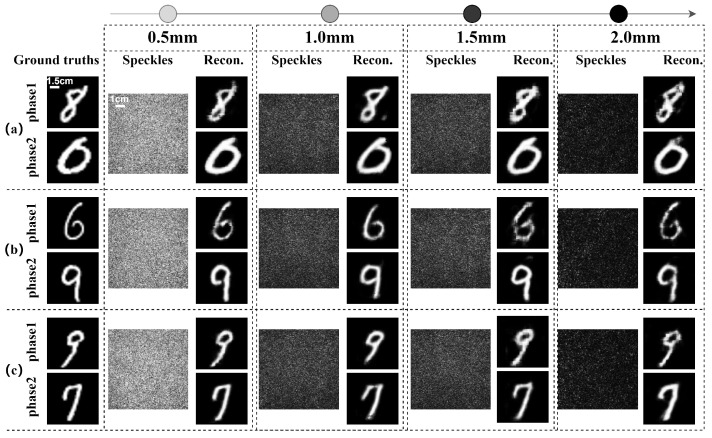
Reconstruction of dual-phase images of orthogonal polarization components using the Trans-CNN network passing through chicken breast tissues with different thicknesses. Three various sets of test datasets are adopted in (**a**), (**b**) and (**c**), respectively.

The multi-channel features were separated into two distinct channels in the final layer of the network’s decoding path, and each channel corresponded to the phase information of one of the two orthogonal polarization components, respectively. The experiments were conducted using four different thicknesses of chicken breast tissue: 0.5 mm, 1.0 mm, 1.5 mm, and 2.0 mm. The phase informations carried by the orthogonal polarization components were significantly affected by the scattering interference of chicken breast tissues with varying thicknesses, resulting in the formation of speckle patterns. However, the phase information of the two orthogonal polarization components can be accurately reconstructed by employing the improved Trans-CNN network, as shown in Figure 5.

To quantitatively assess the reconstruction results, the evaluation metrics of the Pearson correlation coefficient (PCC), structural similarity index (SSIM), and peak signal-to-noise ratio (PSNR) were employed for the reconstructed images. The results are summarized in Table 2. The PCC, PSNR, and SSIM values for the reconstructed images with vector optical fields were significantly higher than those from scalar optical fields, as shown in Table 2a. This indicates that vector optical fields achieve greater image fidelity and superior reconstruction performance after traversing the scattering medium. The quantitative evaluations further demonstrate that the Trans-CNN network model outperforms the conventional CNN in terms of the PCC, SSIM, and PSNR as shown in Table 2a, indicating the significance of integrating the global feature extraction of Transformer architecture.

In the reconstruction of single- and dual-phase images with vector optical fields, the PSNR and SSIM values progressively decrease with the increasing thickness of the scattering medium, as shown in Table 2b. The thicker the medium, the higher the information loss during the transmission of the vector optical field through the medium, ultimately degrading the quality of the reconstructed images. Overall, as the thickness of the chicken breast tissue slices increases, the quality of the image reconstruction shows a declining trend. However, due to potential significant variations in the biological microscopic structures of chicken breast tissue slices of different thickness samples, the reconstructed images retain certain errors, such as the PCC parameter not necessarily decreasing for the two 1.0 mm and 0.5 mm thick samples (see Table 2b). The imaging recovery through a biological tissue using vector optical fields can achieve a medium depth of up to 2.0 mm, demonstrating a significant advantage over other imaging algorithms in terms of imaging depth [37]. Compared to our previous work [24], this work innovatively leverages the evolutions of two orthogonal polarization components of vector optical fields in anisotropic biological media and deep-learning technology, significantly enhancing the robustness of imaging reconstruction in anisotropic and dynamic time-varying biological scattering media. The hybrid network model integrates convolutional neural networks (CNN) and a Transformer architecture. The CNN extracts local features of a speckle image, while the Transformer captures global features, enabling adaptive vectorial restoration of dynamically time-varying speckle patterns. The experimental results demonstrate the excellent robustness and generalization capabilities of the model for reconstructing the two orthogonal polarization components from dynamic speckle patterns behind anisotropic biological media. This capability enables the simultaneous reconstruction of the different phase information carried by the two orthogonal polarization components in the vector optical field from a speckle pattern, exploring further practical applications in materials science, biomedical research, environmental monitoring, and industrial inspection.

## 4. Discussion

Biological tissue imaging faces significant challenges due to the anisotropic nature and complex scattering properties of soft tissues. The inhomogeneous microstructure—comprising muscle fibers, fat, and connective tissues—induces spatially varying scattering characteristics. Unlike isotropic scattering media (e.g., ground glass), where two orthogonal polarization components propagate independently (i.e., $T_{12}\left(m,n,u,v\right)=T_{21}\left(m,n,u,v\right)=0$ [24,29]), biological tissues exhibit polarization-dependent scattering, where these components undergo reciprocal conversion ($T_{12}\left(m,n,u,v\right)\neq 0$ and $T_{21}\left(m,n,u,v\right)\neq 0$). This makes polarization evolution in biological tissues highly complex. Furthermore, dynamic variations such as tissue deformation caused by water loss or gravitational forces further complicate image reconstruction.

Traditional speckle interferometry has been widely used in experimental mechanics to analyze object deformation or displacement based on interference patterns [38]. However, its effectiveness in biological tissue imaging is limited by phase distortion, non-uniform intensity distributions, and image quality degradation due to dynamic scattering. While long-exposure speckle interferometry can mitigate some of these issues [39], it is not well-suited for real-time imaging of biological tissues. In contrast, deep learning-based speckle pattern analysis methods have demonstrated significant advantages in descattering, noise suppression, and robust image reconstruction. By leveraging large datasets, deep learning models can overcome the limitations of traditional methods, providing superior robustness and generalization capabilities in complex scattering media.

Compared to our previous work [24], this study innovatively leverages the evolution of two orthogonal polarization components of vector optical fields in anisotropic biological media and deep-learning technology, significantly enhancing the robustness of imaging reconstruction in anisotropic and dynamic time-varying biological scattering media. The hybrid network model integrates convolutional neural networks (CNNs) and a Transformer architecture. The CNN extracts local features of a speckle image, while the Transformer captures global features, enabling adaptive vectorial restoration of dynamically time-varying speckle patterns. The experimental results demonstrate the excellent robustness and generalization capabilities of the model for reconstructing the two orthogonal polarization components from dynamic speckle patterns behind anisotropic biological media.

Beyond its application in machine reading of patterns on biological tissues, our method holds promise for broader biological structure evaluation. By utilizing vector light fields to extract spatially resolved polarization state information, this approach can be extended to characterize tissue anisotropy, microstructural variations, and birefringence properties—key factors in histopathological analysis and disease detection. Potential applications include imaging and quantifying collagen fiber distributions in connective tissues, identifying microstructural abnormalities in cancerous tissues, and assessing dynamic physiological changes in living organisms. Furthermore, integrating this method with other imaging techniques such as optical coherence tomography (OCT) and fluorescence imaging could provide a more comprehensive assessment of biological structures at multiple scales. These advancements demonstrate the potential of our approach for medical diagnostics, tissue engineering, and biomedical research.

## 5. Conclusions

This study proposes a novel Trans-CNN network architecture for scattering imaging reconstruction in dynamic time-varying biological tissues with vector optical fields. The experimental results demonstrate that the utilization of two orthogonal polarization components of vector optical fields significantly enhances the robustness of scattering imaging reconstruction against complex dynamic time-varying scattering environments, due to an additional dimension describing the properties of an optical field. The incorporation of CNN with Transformer networks enhances the model’s ability to capture both local and global information during the reconstruction process, achieving adaptive vectorial-restoration of dynamic time-varying speckle patterns. The Trans-CNN network model can simultaneously reconstruct the distinct phase images carried by two orthogonal polarized components of the vector optical field from dynamic time-varying speckle patterns. This approach aids in the vectorial restorations of dynamically scattering imaging in biological tissues and expands its application in fields such as materials science, biomedical research, environmental monitoring, and industrial inspection.

## Figures and Tables

**Table 1:** Average validation values of the corresponding image reconstruction results for the temporal evolution of speckle image variations.

	$T_{0}$	$T_{1}$	$T_{2}$	$T_{3}$
PCC	0.9824	0.9876	0.9835	0.9805
SSIM	0.8497	0.8523	0.8477	0.8461
PSNR (dB)	33.1721	33.5794	33.0961	33.2767

**Table 2:** Average validation values of single- and dual-phase image reconstruction results with scalar and vector optical fields through chicken breast tissue slices.

(a)	Trans-CNN and CNN Networks Reconstruction				(b)	Single-Phase Image Reconstruction			Dual-Phase Image Reconstruction		
		PCC	SSIM	PSNR (dB)		PCC	SSIM	PSNR (dB)	PCC	SSIM	PSNR (dB)
Scalar	Trans-CNN	0.9728	0.6715	32.5661	0.5 mm	0.9626	0.8768	34.3944	0.9719	0.8608	35.5563
	CNN	0.4196	0.5876	28.6154	1.0 mm	0.9886	0.8529	33.1625	0.9424	0.7112	34.5598
Vector	Trans-CNN	0.9886	0.8529	33.1625	1.5 mm	0.6422	0.6149	30.5346	0.5129	0.6644	34.1004
	CNN	0.7073	0.6282	29.3095	2.0 mm	0.6498	0.5598	30.4740	0.5272	0.6285	33.6649

## Data Availability

Data underlying the results presented in this paper are not publicly available at this time but may be obtained from the authors upon reasonable request.
